# Schizophrenia and Bipolar Illness in the Relatives of University Scientists: An Epidemiological Report on the Creativity-Psychopathology Relationship

**DOI:** 10.3389/fpsyt.2019.00175

**Published:** 2019-04-02

**Authors:** Josef Parnas, Karl Erik Sandsten, Claus Høstrup Vestergaard, Julie Nordgaard

**Affiliations:** ^1^Mental Health Center Glostrup, Broendby, Denmark; ^2^Center for Subjectivity Research, University of Copenhagen, Copenhagen, Denmark; ^3^Early Psychosis Intervention Center, Roskilde, Denmark; ^4^Research Unit for General Practice, Department of Public Health, Aarhus University, Aarhus, Denmark; ^5^Mental Health Center Amager, Copenhagen, Denmark

**Keywords:** academics, schizophrenia, bipolar, creativity, vulnerability

## Abstract

A potential link between creativity and mental illness has been a longstanding topic for human studies and empirical research. The major problem is defining creativity and establishing its measurable indicators. A few high-quality epidemiological studies have been undertaken and point to a link between creativity and vulnerability to mental illness. Demonstrating such a shared vulnerability could expand our understanding of mental illnesses and open up new avenues of empirical research. In this epidemiological study, we defined scientists (academics) at the universities as individuals assumed to exhibit “more creativity” than the background population. In a register coupling with a population of 588,532 people, we examined successful university academics' first- and second-degree relatives for diagnosed mental disorders and compared those figures with controls from the background population controlling for educational level. The relatives of the academics had significantly increased risk of suffering from schizophrenia or bipolar disorder. For bipolar disorder, it is perhaps temperamental features and high energy levels that contribute to this association. In the case of schizophrenia, the mediating bridge may involve an amplification of human tendency to question the obvious and “taken-for-granted.” Creativity and an increased risk for mental disorders seem to be linked by a shared vulnerability that is not manifested by clinical mental disorders in the academics.

## Introduction

The relationship between creativity and mental illness has been a topic of human studies for centuries, and more recently for empirical research as well (1). For a succinct presentation of the evolution of methodologies see (2). It is being debated whether creative abilities are general in nature or domain specific (3) and there are multiple theories on the cognitive and biological processes involved in creativity (4). In a common-sense view, creativity is understood as an ability or process involved in the creation of something novel, original, and valued. In a review article creativity is defined in a similar way as involving “…the production of novel, useful products” (5). Thus, the ultimate validation whether a given psychological process was creative or not is testified by the achievement. In other words, declaring someone for being creative is a *post hoc* judgement.

In the context of empirical research there are many unresolved conceptual problems (4). These include the definition of creativity and the conceptualizations of creative processes which are all a matter of ongoing debates: “Reviews of the existing literature […] have shown that the field is heavily fragmented and its neuroscientific findings are invalidated by false category formations and compound constructs.” (4).

The research literature is far from an agreement on the relationship between creativity and vulnerability to mental disorder (1). Studies that report a positive association depict such associations as an inverted U, i.e., vulnerability to or low levels of psychopathology are associated with creative abilities which sharply decline with increasing levels of psychopathology (6, 7).

From our particular interest in schizophrenia (8) we will emphasize four important epidemiological studies. Karlsson (9) examined relatives of schizophrenia patients in Iceland and compared them with a control population and found around twice as many in artistic professions among the biological relatives of schizophrenia patients. Kyaga et al. performed a register study in Sweden searching for individuals with psychiatric diagnoses and coupled these data with census information of self-reported occupational status (10). They found that individuals with bipolar disorder and healthy siblings of people with schizophrenia or bipolar disorder were overrepresented among the scientific and artistic professions. In a subsequent register study of another patient sample Kyaga et al. replicated their previous results (11). In addition, they found that individuals with self-reported creative professions were not more likely to suffer from mental disorders except for bipolar disorder.

Recently, Powers et al. studied around 25% of the population of Iceland (86,000). On the basis of the polygenetic schizophrenia risk score they identified 6% treated schizophrenia patients and 2% of members of artistic organizations. The findings were statistically highly significant. They conclude: “Thus, the main finding presented here is that creativity, conferred, at least in part, by common genetic variants, comes with an increased risk of psychiatric disorders conferred by the same genetic variants” (12).

The observed link between creativity and bipolar illness is perhaps due to temperamental features such as high energy and activity levels and the propensity to rapid and combinatorial thinking (13). In the case of schizophrenia there may be envisaged several mechanisms such as a propensity to non-ordinary cognitive styles [e.g., the so called “divergent thinking” (14)] and/or subtle experiential changes expressed in the schizotypal phenotype (15). Creative abilities seem to involve activity in specific neural networks (16).

Our own (17–20) and others [for a review, see (15)] research suggests that it is a spectrum of disorders with varying manifestations but with a certain fundamental phenotypic core gestalt shared by manifest schizophrenia and attenuated schizotypy (21–24). In a phenomenological perspective (8, 24) this core gestalt, i.e., the most elementary phenotypic vulnerability manifestation to schizophrenia consists in a disorder of pre-reflective attunement between the self and the world. This involves instability in the basic self with a tendency to solipsistic experiences of another ontological dimension and profound insight. It also involves the loss of “common sense,” i.e., an experience of the lack of naturalness and obviousness of the world. Moreover, self-disorders seem to be associated with certain alterations of imaginative processes (25–27). Jointly, these experiential changes may imply a propensity to hyperreflectivity, experiences of enlightenment and a tendency to question what ordinary people take for granted. This is perhaps an aspect of human experience (28) that may provide here a bridge between schizophrenia and creativity. Thus, we hypothesize that vulnerability to schizophrenia will be associated with an increased ability to novel and original thoughts, feelings, and expressions.

On the basis of these considerations we decided to study the frequency of schizophrenia and other mental illnesses among the relatives of successful academics i.e., scientists employed in tenured positions at Danish universities. We assumed that such population would reflect a quasi-objective creative achievement compared to the background population. Thus, we hypothesized an increased risk for schizophrenia spectrum disorders among biological relatives of successful university academics.

## Materials and Methods

### Population

We designed a study with elements from both matched cohort studies as well as case-control studies. We received information on all academic-scientific employees in tenured positions at three main Danish universities (Copenhagen University, Aarhus University, and the University of Southern Denmark in Odense), in total 11,803 individuals [hereafter referred to as “Academics” (A)]. At birth, all Danish citizens are assigned a unique personal identification number in the Danish Civil Registration System (29). Via the register the Academics were matched 1:6 on age, gender and municipality of residence with randomly selected controls (C) from the background population. The Danish Civil Registration System allowed us to identify first- and second- degree relatives of the Academics (RA) and controls (RC).

In order to track familiar transmission patterns, we divided this population into five subgroups: children, parents, grandparents, siblings, and nephews/nieces. Grandchildren were also identified, but ultimately excluded from analysis due to the very low age.

### Outcome

The Danish Psychiatric Central Research Register holds information on all admissions to psychiatric hospitals from 1969 and outpatient contacts from 1995 and onwards (30). From this register we received information on psychiatric diagnoses on Academics, controls and their relatives (A, RA, C, RC).

For the whole sample, we collected all psychiatric diagnoses and classified each person into one of six diagnostic outcome groups: Schizophrenia, non-affective psychosis, bipolar disorder, melancholia, any other mental disorder, or no psychiatric diagnosis. We imposed a hierarchy following ICD-10 using the methods of other register studies (10, 31).

### Covariates

RA and RC were not matched (only the Academics and Controls were), thus necessitating adjustment for age and gender. Furthermore, we wished to adjust for intelligence level as it has been shown to be the most important epidemiological risk factor for schizophrenia (32, 33) and therefore constitutes a confounder. We used educational level as a proxy for intelligence. Information on educational level was provided by Statistics Denmark. We were not able to obtain information on Faculty membership of the Academics.

### Analyses

The five subgroups of relatives were analyzed in identical fashion; in a logistic model with “relation to Academics or control” as the dependent variable and the six outcome types as the independent variable, adjusted for education level, gender, and age. Adjustment for the latter was done by way of a cubic spline, in order to obtain a good adjustment while spending only a few degrees of freedom. The A and C were analyzed separately from the relative subgroups.

## Results

The population of the study is presented in Table 1. The Odds Ratio (OR) for the Academics to be diagnosed with any mental disorder was 0.440 (*p* < 0.0000; 95% CI 0.398–0.485), for bipolar disorder 0.434 (*p* < 0.0007; 95% CI 0.269–0.702), and for schizophrenia 0.167 (*p* < 0.0000; 95% CI 0.108–0.257).

**Table 1:** The study population.

	**Academics**	**Control**	***P*-value**
**Casus (*****N*****)**	11, 805	70, 818	
Male	6,894 (58.4%)	41,364 (58.4%)	0.683
Mean date of birth	Aug 1972 (12.0years)	Aug 1972 (12.0years)	0.686
**Children (*N*)**	16, 398	84, 459	
Male	8,526 (52.0%)	43,253 (51.2%)	0.067
Mean date of birth	Dec 2000 (11.7years)	Dec 1998 (12.7years)	0.000
**Parents (*N*)**	15, 422	91, 929	
Male	7,642 (49.6%)	45,363 (49.3%)	0.440
Mean date of birth	Apr 1943 (11.6years)	Jul 1945 (11.8years)	0.000
**Grandparents (*N*)**	7, 466	48, 683	
Male	3,651 (48.9%)	23,788 (48.9%)	0.660
Mean date of birth	Dec 1923 (9.3years)	Jan 1927 (9.8years)	0.000
**Siblings (all) (*N*)**	12, 517	88, 593	
Male	6,479 (51.8%)	45,878 (51.8%)	0.667
Mean date of birth	Oct 1974 (11.5years)	Apr 1975 (12.0years)	0.000
**Maternal half-siblings (*N*)**	1, 415	12, 246	
Male	729 (51.5%)	6, 209 (50.7%)	0.389
Mean date of birth	Jan 1979 (13.5years)	Jul 1979 (13.7years)	0.161
**Paternal half-siblings (*N*)**	636	9, 347	
Male	323 (50.8%)	4, 756 (50.9%)	0.668
Mean date of birth	May 1978 (11.6years)	Aug 1978 (12.5years)	0.564
**Siblings (share both parents) (*N*)**	10, 466	67, 000	
Male	5,427 (51.9%)	34,913 (52.1%)	0.435
Mean date of birth	Dec 1973 (11.0years)	Jan 1974 (11.3years)	0.444
**Niece/nephew (*N*)**	17, 522	122, 920	
Male	9,015 (51.4%)	63,091 (51.3%)	0.528
Mean date of birth	Feb 2002 (9.8years)	Aug 2000 (10.4years)	0.000
**Total (*N*)**	81, 130	507, 402	

*Numbers, relation to Academics/controls, gender, and mean date of birth*.

**Figure 1:**
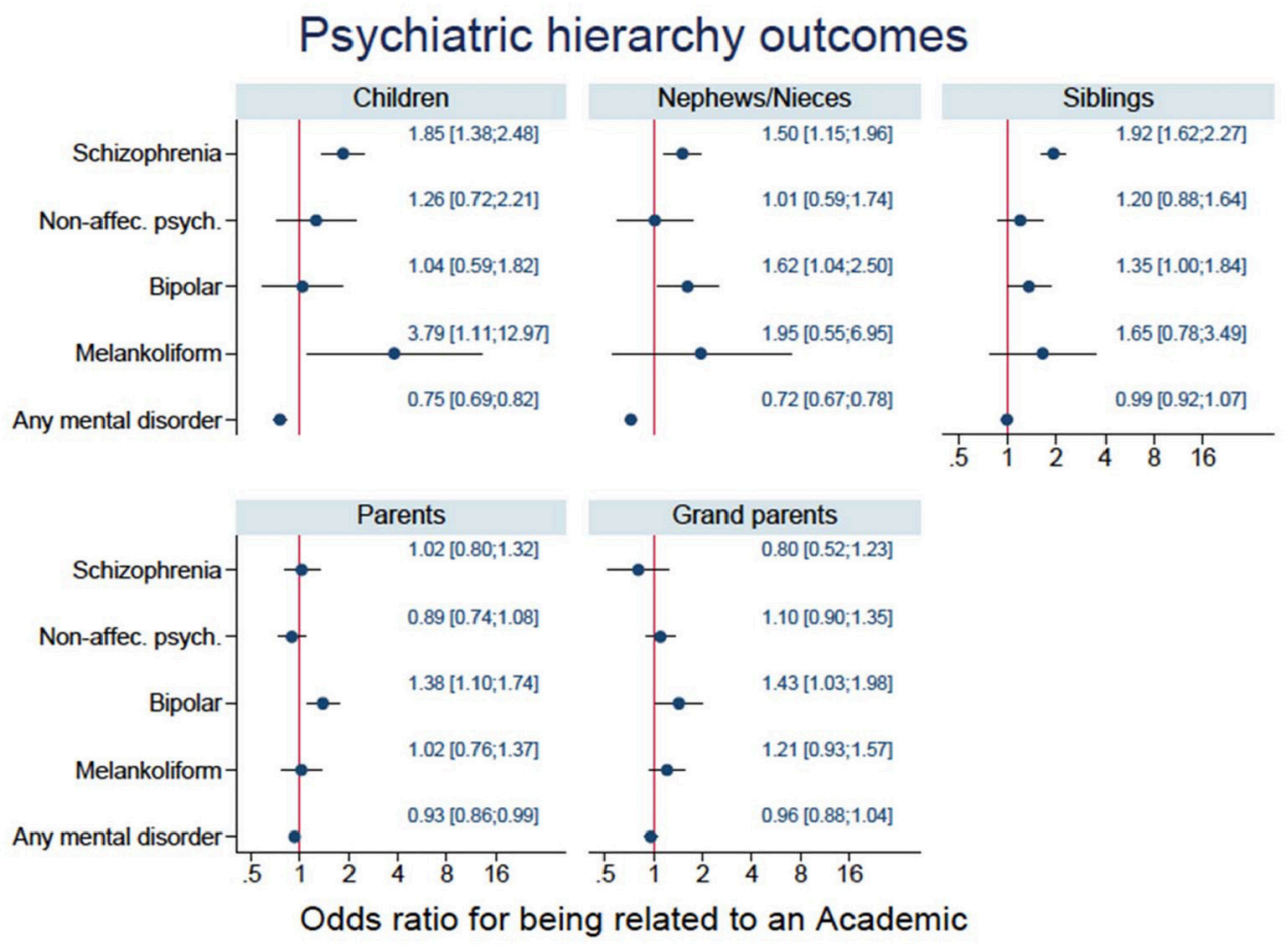
Forest plots for the five subgroups of relatives for the four diagnostic groups and any mental disorder.

The Forest plots in Figure 1 indicate that there was a significant (*p* < 0.05) increased risk for schizophrenia among siblings, children, and nephews/nieces of the Academics. For bipolar disorder the OR was significantly (*p* < 0.05) increased for the academics' parents, grandparents, and nephews/nieces. Furthermore, the OR for the Academics' siblings was borderline significant (*p* = 0.05) for bipolar disorder. Additionally, we analyzed the siblings divided into maternal half-siblings and paternal half-siblings. We found that the OR for maternal half-siblings were significantly increased for schizophrenia however, that was not the case for paternal half-siblings.

## Discussion

We focus our discussion on the relationship between our finding and the epidemiological and genetic studies mentioned in the introduction. It is beyond our scope to address other scientific approaches linking creativity and psychopathology, notably the flourishing neurobiological research.

This study shows that whereas successful academics as a group are less prone to mental disorder than the background population there are increased rates of schizophrenia and bipolar illness among their biological relatives. The study was based on the assumption that successful university academics as a group are more creative than the background population. Obtaining a tenured position at a Danish university is highly competitive and the academic promotion is based on standard academic evaluation criteria. However, this starting assumption may be criticized. Contemporary university career is very much framed by bureaucratic influences and being a researcher is no longer reserved for the few, truly fascinated by their scientific field, but has become much more akin to a standard type of job (34). Western universities have become increasingly invaded by corporate-managerial culture and academic career has become very much dependent on the quantity rather than the quality of publications. French sociologist Pierre Bourdieu (35) distinguished between “eternal” and “temporal” scientists. The eternal scientist is a sort of ideal type researcher fascinated by and digging ever deeper in his topic and emitting power through his reputation and esteem. The temporal scientist makes his career through networking, political engagements, committee and funding agencies, membership etc. Given the corporate managerial shift of the academic culture one could argue that the proportion of the temporal type of scientist has increased dramatically and thereby undermining our starting assumption of higher levels of creativity in our sample of academics. Moreover, we have to acknowledge that the findings of the association between the academic-scientific status and increased rates of schizophrenia and bipolar disorder in the relatives may be caused by multiple other unknown factors unrelated to creativity. While we acknowledge these reservations we still believe that our sample of Danish scientists reflect more creativity compared with the background population. It should be emphasized that this study was solely undertaken on the specific basis of our hypothesis.

Importantly, our index group is characterized by possessing a quasi-objective scientific achievement. Our study, being epidemiological in nature lacks the information about the individual indicators of creativity among the study population.

The risk of any mental illness among the Academics were significantly lower than in the controls probably because an academic career is too demanding for people with mental disorders that are associated with a hospital contact. Thus, in this particular sample individuals with hospitalization-demanding disorder have been selected away. We failed to demonstrate an increase of manifest schizophrenia among the parents of Academics most likely because having a parent with early onset chronic mental illness reduces ones chances of a smooth educational career (36).

Our findings are concordant with the studies referred to in the introduction (9–12). Our study design resembles those of Kyaga et al. (10, 11) in being case control studies using creative professions as a creativity proxy. However, our study does not start with a psychiatric patient population, but rather with a non-clinical sample assumed to be more creative than the general population (successful academics). This design makes it difficult to compare minutiae of ours and Kyaga's results. However, all these studies support the notion of a link between creativity and the vulnerability to mental illness. The results are compatible with a shared polygenetic susceptibility to schizophrenia, bipolar illness, and creativity. Beyond a certain point the increase of liability will begin to impede with creative abilities and lead to manifest psychopathology and a status as a psychiatric patient (7). What seems apparent is that the hypothesized relationship between creativity in academics and the increased risk for schizophrenia and bipolar disorder in their relatives must be mediated by a vulnerability that is not manifested by overt mental disorder in the academics, consistent with the inverted U-curve model (6, 7).

## Data Availability

The datasets for this study will not be made publicly available because it is prohibited by the Danish Act of Privacy.

## Ethics Statement

The study protocol was reviewed and accepted first by the dean of the Faculty of Health and Medical Sciences, University of Copenhagen and subsequently by the administrations of all three universities. The study was approved by the Danish National Committee on Health Research Ethics. All data were anonymized and the authors had no access to any personal or other data that could identify individuals.

## Author Contributions

JP and JN designed and planned the study and wrote the first draft of the paper. KS and CV did the statistical analyses. All authors have contributed to and approved the final manuscript.

### Conflict of Interest Statement

CV was supported by an unrestricted grant from the Lundbeck Foundation (grant number: R155-2012-11280). The remaining authors declare that the research was conducted in the absence of any commercial or financial relationships that could be construed as a potential conflict of interest.
